# Molecular identification of yeast communities isolated from nail specimens by PCR-RFLP and PCR-FSP methods

**DOI:** 10.22034/cmm.2024.345237.1539

**Published:** 2024-05-07

**Authors:** Ahmad Jabrodini, Mitra Zaighami, Ali Khodadadi, Keyvan Pakshir, Hasti Nouraei, Hossein Khodadadi

**Affiliations:** 1 Department of Medical Parasitology and Mycology, School of Medicine, Shiraz University of Medical Sciences, Shiraz, Iran; 2 Faculty of Medicine, Najafabad Branch, Islamic Azad University, Najafabad, Isfahan, Iran

**Keywords:** Molecular identification, Onychomycosis, PCR-RFLP, PCR-FSP, Yeast communities

## Abstract

**Background and Purpose:**

Onychomycosis is a common fungal infection that affects the nails, caused by various fungal agents. Moreover, yeast onychomycosis has increased in recent years. Yeast isolates might not be identified at the species level by conventional methods, whereas molecular methods can identify yeast isolates more accurately. This study aimed to identify yeast communities isolated from nail specimens by polymerase chain reaction (PCR)-restriction fragment length polymorphism (RFLP) and PCR- fragment size polymorphism (FSP) methods.

**Materials and Methods:**

This experimental study was conducted on archival yeast isolates obtained from 269 patients suspected of onychomycosis who referred to the Medical Mycology Laboratory at Shiraz University of Medical Sciences in Shiraz, Iran, between April 2022 and March 2023. Onychomycosis was confirmed through direct examination and culture of nail specimens. The PCR-RFLP and PCR-FSP methods were used to identify yeast isolates.

**Results:**

In total, 78 (28.99%) yeast strains were identified. *Candida albicans* was the most common species, followed by *Candida parapsilosis* complex and *Candida tropicalis*. Uncommon species of yeasts, such as *Candida utilis*, *Candida pararugosa*, *Candida nivariensis*, and *Rhodotorula rubra* were identified by molecular methods. The PCR-FSP method showed a strong agreement with the PCR-RFLP method in the identification of common yeast agents causing onychomycosis (κ=0.84).

**Conclusion:**

It seems necessary to use molecular diagnostic tools in addition to conventional methods to identify yeast isolates in clinical laboratories. The rapid and accurate identification of fungal agents causing onychomycosis is useful for the selection of an appropriate treatment strategy.

## Introduction

Onychomycosis is a fungal infection of the nails. The infection causes discoloration, thickening, and disfiguration of the nail plate [ 1 ]. Onychomycosis is the most common nail disease, responsible for approximately 50% of all nail disorders [ 2 ]. The prevalence of this infection is estimated at 5.5% of the world population [ 3 ].

Although onychomycosis is not life-threatening, it often causes psychosocial effects, occupational discomfort, and a high cost of treatment, which affects the quality of life and the beauty of the hands and feet of the patient [ 4 ]. Moreover, it can be a serious health-threatening problem for immunocompromised hosts. Some factors related to the increase in the prevalence of onychomycosis include age, occupation, weather, lifestyle, repeated nail trauma, diabetes, immune system dysfunction, use of broad-spectrum antibiotics, and nowadays, some unhealthy manipulation of the nails during manicures or pedicures [ 5 , 6 ].

The most prevalent causative agents of onychomycosis are dermatophytes, non-dermatophyte molds, and yeasts (mostly species of *Candida*). A growing incidence of yeast onychomycosis has occurred in recent decades [ 7 ].

Conventional methods, such as direct microscopic examination of nail scrapings and culture are used in the diagnosis of onychomycosis [ 8 ]; however, with these methods, it is usually not possible to identify fungal pathogens at the species level [ 5 ]. Due to the difference in susceptibility to antifungal drugs among different fungal genera and species, identification at the species level is important to manage a correct therapeutic strategy [ 9 ]. 

Different molecular methods, including polymerase chain reaction (PCR)-sequencing, real-time PCR, PCR-restriction fragment length polymorphism (RFLP), and PCR-fragment size polymorphism (FSP), allow the discrimination of fungal pathogens at the species level [ 10 , 11 ]. Internal transcribed spacer 1 (ITS1) and ITS2 regions are widely used for species-level identification due to the high degree of variability among fungi [ 12 ]. In this regard, the present study aimed to identify yeast communities isolated from nail specimens by PCR-RFLP and PCR-FSP methods.

## Materials and Methods

### Study population and yeast isolates

This experimental study was conducted on archived yeast isolates collected from 269 patients suspected of onychomycosis. Samples and yeast isolates had been archived in the Medical Mycology Laboratory at Shiraz University of Medical Sciences in Shiraz, Iran, between April 2022 and March 2023.

A direct microscopic examination of nail specimens was carried out using a 20% potassium hydroxide (KOH) solution. A portion of the nail specimens were cultured on Sabouraud dextrose agar (SDA; Merck, Germany) supplemented with chloramphenicol (0.05 mg/ml) and incubated at 35 °C for 1 week. Cultures were checked on daily. CHROM-agar *Candida* medium (CHROMagar^TM^, Paris, France) was used for the initial identification of *Candida species*.

Yeast reference strains, including *Candida albicans* American Type Culture Collection (ATCC) 10231, *Candida glabrata* ATCC 2001, *Candida tropicalis* ATCC 750, *Candida parapsilosis* ATCC 22019, *Candida auris* CentraalBureau voor Schimmelcultures (CBS) 12372, *Candida haemulonii* CBS 7801, and *Saccharomyces cerevisiae* Persian Type Culture Collection (PTCC) 5177, were used in the PCR-RFLP and PCR-FSP methods as controls to study size variation. In order to obtain fresh colonies, yeast reference strains were cultured on SDA. The culture media were incubated at 35 °C for 24-72 h.

This study was approved by the Research Ethics Committee of Shiraz University of Medical Sciences (IR.SUMS.REC.1397.727).

### Molecular identification of yeast isolates

The DNA from all yeasts was extracted using the saturated lithium acetate method as described previously [ 13 ]. To identify yeast isolates, the previously described PCR-RFLP and PCR-FSP methods were performed [ 14 ]. For the identification of yeast isolates by PCR-RFLP method, at the first step, the ITS1–5.8S–ITS2 region was amplified by using universal primers ITS1 (5'-TCCGTAGGTGAACCTGCGG-3') and ITS4 (5'-TCCTCCGCTTATTGATATGC-3'). In the second step, all PCR products were digested with the restriction enzyme *Msp*I for 2 h at 37 °C according to the method already described [ 6 ]. Finally, identification of the yeast isolates was performed by comparison of the electrophoretic band sizes of the PCR-RFLP products with the specific PCR-RFLP patterns described previously [ 9 ] (Table 1).

**Table 1:** Size of ITS region after digested with *Msp*I in PCR-RFLP reaction and size of ITS1 and ITS2 fragments in PCR-FSP according to the yeast strains

Yeast strain	Mean Length of ITS1-5.8S-ITS2 region	Length of fragments after enzymatic digestion with *Msp*I in PCR-RFLP	Length of ITS1 and ITS2 fragments in PCR-FSP	
			ITS1	ITS2
*Candida albicans*	537	239, 298	219	338
*Candida tropicalis*	526	186, 340	218	327
*Candida glabrata*	881	320, 561	482	419
*Candida kefyr*	720	720	309	432
*Candida krusei*	510	250, 260	182	347
*Candida orthopsilosis*	510	510	220	311
*Candida parapsilosis*	530	530	220	310
*Candida metapsilosis*	531	531	236	314
*Candida utilis*	565	565	220	364
*Candida nivariensis*	760	205, 236, 319	188	326
*Candida pararugosa*	415	-	164	271
*Rhodotorula rubra*	608	503, 105	232	404

PCR: polymerase chain reaction, RFLP: restriction fragment length polymorphism, ITS: internal transcribed spacer, FSP: fragment size polymorphism

In order to identify yeast isolates by the PCR-FSP method, the yeast ITS1 and ITS2 regions were used as targets to simultaneously amplify in separate reaction tubes. The ITS1 region was amplified using ITS1 and ITS2 (5'-GCTGCGTTCTTCATCGATGC-3') primers. The ITS3 (5'-GCATCGATGAAGAACGCAGC-3') and ITS4 primers were used for amplification of the ITS2 region. Yeast isolates were identified according to the electrophoretic two-band size pattern as described before [ 14 ] (Table 1).

### Statistical analysis

Chi-squared and Fisher's exact tests were used for statistical analysis in SPSS software (version 20). The variables were presented as numbers and percentages. A statistical significance level of P ≤ 0.05 was considered.

## Results

Out of 269 suspected onychomycosis patients, 78 (28.99%) yeast agents were isolated. *Candida albicans* complex (*C. albicans*, *C. dubliniensis*, and *C. africana*) (n=39, 50%, green colony), *C. tropicalis* (n=10, 12.82%, blue colony), *C. glabrata* (n=7, 8.97%, pale purple/mauve colony), and *C. krusei* (n=3, 3.84%, pink colony) were identified by culture on CHROM-agar *Candida*. Moreover, 19 (24.35%) yeast isolates did not show color change in the colony (Table 2).

**Table 2:** Identification of yeast isolates causing onychomycosis by CHROM-agar *Candida* medium, PCR-RFLP, and PCR-FSP methods

Yeast strains	CHROM-agar	PCR-RFLP	PCR-FSP
*C. albicans*	-	39	40
*C. albicans complex*	39	-	-
*C. tropicalis*	10	10	7
*C. parapsilosis*	-	-	6
*C. orthopsilosis*	-	-	7
*C. metapsilosis*	-	-	2
*C. parapsilosis complex*	-	14	-
*C. glabrata*	7	6	3
*C. kefyr*	-	3	3
*C. krusei*	3	3	2
*C. utilis*	-	-	2
*C. nivariensis*	-	-	1
*C. pararugosa*	-	-	1
*Rhodotorula rubra*	-	-	2
Unknown Yeast isolates	19	3	2
Total	78	78	78

**Figure 1:**
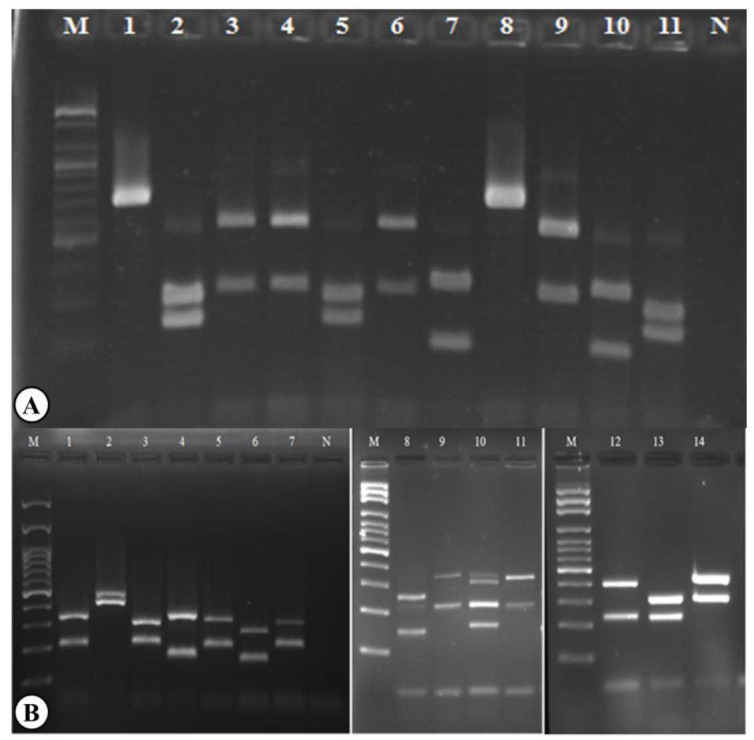
A) Agarose gel electrophoretic band pattern of polymerase chain reaction-restriction fragment length polymorphism products of yeast isolates; Lane M: 100-bp DNA ladder; Lanes 1 and 8: *C. kefyr* (720 bp); Lanes 2, 5, and 11: *C. albicans* (239, 298 bp); Lanes 3, 4, 6, and 9: *C. glabrata* (320, 561 bp); Lanes 7 and 10: *C. tropicalis* (186, 340 bp); Lane N: negative control. B) Agarose gel electrophoresis pattern of PCR-FSP products of yeast isolates; Lane M: 100-bp DNA ladder; Lane 1: *C. albicans* (219, 338 bp); Lane 2: *C. glabrata* (419, 482 bp); Lane 3: *C. parapsilosis* (220, 310 bp); Lane 4: *C. krusei* (182, 347 bp); Lane 5: *C. tropicalis* (218, 327 bp); Lane 6: *C. pararugosa* (164, 271 bp); Lane 7: *C. orthopsilosis* (220, 311 bp); Lane N: negative control; Lane 8: *C. pararugosa* (164, 271 bp); Lane 9: *C. utilis* (220, 364 bp); Lane 10: (mix and unidentified isolates); Lane 11: *C. utilis*(220, 364 bp); Lane 12: *Rhodotorula rubra* (232, 404 bp); Lane 13: *C. orthopsilosis* (220, 311 bp); and Lane 14: *C. kefyr* (309, 432 bp).

According to the PCR-RFLP method, 75 yeast strains, including *C. albicans* (n=39, 50%), *C. parapsilosis* complex (*C. parapsilosis*, *C. orthopsilosis*, and *C. metapsilosis*) (n=14, 17.94%), *C. tropicalis* (n=10, 12.82%), *C. glabrata* (n=6, 7.69%), *C. kefyr* (n=3, 3.84%), and *C. krusei* (n=3, 3.84%), were identified (Figure 1A) (Table 2). It should be mentioned that three yeast isolates (3.84%) were not identified by the PCR-RFLP method.

Based on the PCR-FSP method, *C. albicans* (n=40, 51.28%) was the most frequently isolated yeast strain from patients, followed by *C. tropicalis* (n=7, 8.97%), *C. orthopsilosis* (n=7, 8.97%), *C. parapsilosis* (n=6, 7.69%), *C. glabrata* (n=3, 3.84%), *C. kefyr* (n=3, 3.84%), *C. krusei* (n=2, 2.56%), *C. metapsilosis* (n=2, 2.56%), *C. utilis* (n=2, 2.56%), *Rhodotorula rubra* (n=2, 2.56%), *C. nivariensis* (n=1, 1.28%), and *C. pararugosa* (n=1, 1.28%). Two yeast isolates (2.56%) were not identified by this method (Figure 1B and Table 2).

Identification and differentiation of *Candida parapsilosis* complex species were performed based on a comparison of the length of fragments with both PCR-RFLP and PCR-FSP methods. By comparison of the fragments obtained in the PCR-RFLP method, *C. orthopsilosis* (510 bp) was distinguished from *C. parapsilosis* (530 bp) and *C. metapsilosis* (531 bp). Afterward, *C. parapsilosis* (220 bp) was differentiated from *C. metapsilosis* (236 bp) by comparison of the length of the ITS1 fragment in the PCR-FSP method.

The PCR-FSP method indicated strong agreement with the PCR-RFLP method (κ=0.84). The concordant and discordant results between the PCR-RFLP and PCR-FSP methods are summarized in Table 3.

**Table 3:** Agreement and disagreement rates between the PCR-RFLP and PCR-FSP results

Methods	*Candida* species	PCR-RFLP						Total	Kappa-value
		*C. albicans*	*C. tropicalis*	*C. glabrata*	*C. parapsilosis* complex	*C. kefyr*	*C. krusei*		
PCR-FSP	*C. albicans*	6	1	0	0	0	0	7	Strong
	*C. tropicalis*	0	6	0	0	0	0	6	
	*C. glabrata*	0	0	5	0	0	0	5	
	*C. parapsilosis*	0	0	0	15	0	1	16	
	*C. kefyr*	0	0	0	0	3	0	3	
	*C. krusei*	0	0	0	0	0	2	2	
	*C. utilis*	0	2	0	0	0	0	2	
	*C. nivariensis*	0	0	1	0	0	0	1	
**Total**		**6**	**9**	**6**	**15**	**3**	**3**	**42**	

PCR: Polymerase Chain Reaction, RFLP: Restriction Fragment Length Polymorphism, ITS: Internal Transcribed Spacer, FSP: Fragment Size Polymorphism

## Discussion

Rapid and accurate identification of infecting agents causing onychomycosis is essential for the selection of an appropriate therapeutic strategy and prevention of severe nail damage [ 15 , 16 ]. While PCR-sequencing can accurately detect yeasts, it is necessary to apply alternative molecular methods, such as PCR-RFLP and PCR-FSP, in routine laboratories and countries with limited access to sequencing machines. The PCR-FSP method has demonstrated its superior performance and efficiency in the identification of the most common yeasts, compared to traditional mycology methods [ 14 ]. This study provided appropriate data on the usefulness of an accessible molecular method for the identification of yeasts causing onychomycosis.

The incidence of onychomycosis is influenced by the climate of the region, gender, age, occupation, lifestyle, and daily habits [ 6 ]. Similar to our findings, in other research [ 4 , 17 , 18 ], *Candida albicans* was the most common yeast strain isolated from onychomycosis. However, in some literature [ 6 , 19 , 20 ], *C. parapsilosis* is mentioned as the most prevalent yeast strain causing onychomycosis. On the other hand, some yeast strains, including Rhodotorula rubra, *C. nivariensis* *C. pararugosa*, and *C. orthopsilosis*, are potential pathogens of onychomycosis; however, they are rarely isolated from nail specimens [ 21 ].

Conventional methods do not have enough sensitivity and specificity to correctly identify all yeast pathogens; therefore, they are not able to identify emerging and rare yeast strains, especially uncommon *Candida* species [ 21 , 22 ].

The ITS1 and ITS2 regions are known targets for different genotyping methods to identify yeast pathogens. These regions have enough differences from each other in nucleotide sequences and the number of nucleotides among different yeast species. Therefore, differences in size can be used in molecular methods to identify and separate yeast agents [ 23 ]. Just by considering the size of the ITS1 and ITS2 regions, it is not possible to distinguish all yeast species. Therefore, some species remain unidentified [ 14 ]. However, common yeasts causing onychomycosis are distinguishable by the amplicon size-based method.

Nowadays, regarding the emergence of drug resistance among pathogenic fungi, it is necessary to use practicable molecular approaches to accurately identify the genus and species of fungi for a successful treatment. Due to the lack of access to sequencing methods, the PCR-RFLP method is one of the most practicable methods for the identification of clinical yeast isolates, especially common *Candida* species [ 24 ]. This method is simple, inexpensive, and reliable [ 9 ]. 

Although the PCR-RFLP differentiation capability is determined by the number of cutting sites on the amplified target created by restriction enzymes, selection and addition of enzymes that produce differentiable patterns may improve the approach [ 22 ]. In the present study, some pathogenic *Candida* species, including *C. albicans*, *C. tropicalis*, *C. glabrata*, and *C. krusei*, were identified by the PCR-RFLP method. As mentioned, identification and differentiation of these species from other yeast isolates is important for the selection of appropriate antifungal drugs in the treatment of onychomycosis due to their resistance to some antifungal agents, especially azoles. Similarly, several studies employed the PCR-RFLP method using the ITS1–5.8S–ITS2 gene region to identify yeast isolates causing onychomycosis [ 11 , 15 , 24 ].

In the PCR-FSP method, the inherent differences in the size of the ITS 1 and ITS 2 region amplicons amongst yeast species are the basis for their identification [ 23 ]. The PCR-FSP is an easy, more rapid, and inexpensive method compared to the PCR-RFLP for the identification of medically important and some uncommon yeast species [ 14 ]. In this study, following electrophoresis analysis of ITS1 and ITS2 amplicons obtained by PCR-FSP, emerging and uncommon *Candida* species, such as *C. utilis*, *C. nivariensis*, *C. pararugosa*, *C. orthopsilosis*, *C. metapsilosis*, and *Rhodotorula rubra* were identified. However, this method could not easily distinguish between *C. albicans* and *C. dubliniensis* and also has limited discriminatory power to differentiate species of the *Candida parapsilosis* complex. Therefore, it depends on precautions, such as appropriate electrophoresis and controls [ 10 , 14 ].

The analysis of the concordance between the PCR-RFLP and the PCR-FSP methods in this study indicated a strong concordance between the two methods in the identification of common yeast species causing onychomycosis.

## Conclusion

Findings of this study demonstrated that although the PCR-RFLP method had the ability to identify common clinical yeasts, it had a poor ability to discriminate between emerging and rare yeasts, such as *C. utilis* and *C. nivariensis*. The PCR-FSP method showed a strong ability to identify rare yeasts, such as *C. pararugosa* and *Rhodotorula rubra*. The limitation of both methods is their inability to discriminate against some uncommon yeasts.
